# Hemostasis, endothelial stress, inflammation, and the metabolic syndrome

**DOI:** 10.1007/s00281-017-0666-5

**Published:** 2017-12-05

**Authors:** Gerald Grandl, Christian Wolfrum

**Affiliations:** 1 Institute of Food, Nutrition and Health, ETH Zurich, Schwerzenbach, Switzerland; 2 Institute for Diabetes and Obesity, Helmholtz Zentrum München, Parkring 13, D-85748 Garching, Germany

**Keywords:** Obesity, Metabolic syndrome, Type 2 diabetes, Hemostasis, Platelets, Coagulation, Endothelial stress, Nitric oxide, NO, Reactive oxygen species, ROS, Inflammation

## Abstract

Obesity and the metabolic syndrome (MS) are two of the pressing healthcare problems of our time. The MS is defined as increased abdominal obesity in concert with elevated fasting glucose levels, insulin resistance, elevated blood pressure, and plasma lipids. It is a key risk factor for type 2 diabetes mellitus (T2DM) and for cardiovascular complications and mortality. Here, we review work demonstrating that various aspects of coagulation and hemostasis, as well as vascular reactivity and function, become impaired progressively during chronic ingestion of a western diet, but also acutely after meals. We outline that both T2DM and cardiovascular disease should be viewed as inflammatory diseases and describe that chronic overload of free fatty acids and glucose can trigger inflammatory pathways directly or via increased production of ROS. We propose that since endothelial stress and increases in platelet activity precede inflammation and overt symptoms of the MS, they are likely the first hit. This suggests that endothelial activation and insulin resistance are probably causative in the observed chronic low-level metabolic inflammation, and thus both metabolic and cardiovascular complications linked to consumption of a western diet.

## Introduction

Recent rise in the incidence of obesity has led to a similar increase in the incidence of type 2 diabetes mellitus (T2DM), which constitutes a metabolic disorder affecting more than 10% of the US population [1]. In addition, T2DM is considered to be the fifth-leading cause of death worldwide, and due to its associated co-morbidities, which require life-long chronic treatment, it is one of the most costly diseases for health care systems. Costs directly related to T2DM range from 2% up to 20% of total health care costs, which adds up to approximately $400 billion in the USA, alone [1].

Type 1 diabetes mellitus (T1DM) in contrast to T2DM is normally not associated with obesity, but rather presents an early onset autoimmune disorder, which leads to ablation of beta-cells and loss of insulin secretion. Consequently, T1DM can be defined as a hypoinsulinemic, hyperglycemic state, while T2DM can be defined as a hyperinsulinemic, hyperglycemic state. Hyperglycemia and the associated co-morbidities, which arise as a consequence of chronic high glucose levels, therefore are considered to be common features of both T2DM and T1DM. Both T1DM and T2DM are important risk factors for the development of different types of vascular diseases such as myocardial infarction, stroke, and other types of peripheral vascular diseases (CVDs) [2]. Diabetic patients thus are much more likely to develop heart disease and stroke than individuals without diabetes mellitus. Furthermore, cardiovascular complications are the leading cause of morbidity and mortality among patients with T1DM and T2DM [3].

The metabolic syndrome (MS) consists of several interconnected physiological, biochemical, clinical, and metabolic factors which increase the risk for CVD and T2DM as well as all-cause mortality (reviewed in [4]). Its clinical identification is based on measures of insulin resistance concomitant with elevated plasma insulin levels, visceral adiposity, atherogenic dyslipidemia (consisting of elevated triglycerides and LDL-cholesterol and reduced HDL-cholesterol), endothelial dysfunction, elevated blood pressure, and a hypercoagulable state. It is nowadays well accepted that a pro-inflammatory state is one component of the MS. Moreover, recent studies have shown that an interrelation between inflammation and metabolic abnormalities in T2DM can be a causative factor for vascular damage and it has been suggested that one indicator of these effects might be endothelial dysfunction in conjunction with a pro-coagulant state [5]. Coagulation is the process by which blood clots, with the primary goal of achieving hemostasis. Essential to clot formation is the formation of fibrin, the protein-mesh that forms the physical base of the blood clot [6]. Another central component of the blood clot are platelets, which are small rotund fragments of megakaryocytes that circulate in the blood. They become activated through a variety of coagulatory signals. Upon activation, they become star-shaped, adherent to activated endothelium, fibrin/fibrinogen, and each other, and they release a variety of coagulatory cytokines and enzymes participating in clot formation [7].

Since inflammation is part of an immune response to infection or injury, which crucially involves increased vascular permeability to immune cells, and immune cell invasion of interstitium to execute clearance of pathogens [8], it is not surprising that there is considerable functional overlap between the processes of platelet activation and coagulation, and inflammation. While it is clear that endothelial cells contribute to inflammatory responses, the role of hemostatic processes and in particular platelets in instigating or mediating immune responses has only begun to be elucidated relatively recently [9]. Platelets contribute to inflammatory processes by a variety of small molecule and protein cytokines they release, but they also play an important physical role, in particular in facilitating attachment of immune cells such as neutrophils and in participating in the formation if neutrophil extracellular traps [10, 11].

Interestingly, both T1DM as well as the MS and T2DM constitute an increased risk for cardiovascular disease, present with elevated glucose levels as well as an impaired plasma lipid profile [12], and are thought to be causally linked to inflammatory processes [13]. With that in mind, here we examine reports of increased coagulation and platelet activation, as well as endothelial stress and inflammation, in diabetic states, and look at their temporal dynamics during metabolic disease to infer possible insight into the etiology of metabolic inflammation and the MS.

## Diabetes as a hypercoagulable state

Vascular complications, which are a common feature of T1DM and T2DM can be due to many factors. On the one hand passive diffusion of glucose into endothelial cells can lead to increases in intracellular glucose concentrations, which in turn can cause increased oxidative stress arising from oxidative degradation of glucose metabolites. Similarly, advanced glycosylation endproducts (AGEs), which are the result of intracellular hyperglycemia, have been shown to be involved in mediating vascular damage. Furthermore, hyperglycemia can be the cause for glycation of proteins, which can promote macro- and microvascular damages. Lastly, it has been reported that hyperglycemia leads to a hypercoagulable state [14].

The exact mechanisms which link hyperglycemia and an increased propensity for coagulation are not completely understood. Already in 1979, Jones et al. [15] reported that fibrinogen survival was reduced in diabetic patients in a hyperglycemic state and was improved when euglycemia was achieved. Furthermore, the authors could show that heparin infusion could normalize the fibrinogen kinetics of hyperglycemic patients suggesting that platelet activation is altered. In 1983, Hughes et al. [16] could show that in 37 type 2 diabetic patients with no clinical evidence of retinopathy or vascular disease, approximately half showed hyperactive platelets and that the effect could be restored when euglycemia was achieved. The fact that these studies were conducted in patients who had not shown any presence of overt vascular disease suggested that hypercoagulation might actually be a causal factor in the development of vascular complications. Since then, many studies have reported that hypercoagulation is observed in diabetic patients and often precedes the onset of symptoms related to vascular damage [17–19]. Nevertheless, it was shown that in type 1 diabetic patients with established microvascular angiopathy, an improvement of glycemic control by a strict regime of insulin infusions did not improve some features of platelet function [20]. Furthermore, it was shown that increased platelet activation was observed in diabetic patients with microvascular angiopathy, independent of glycemic control [21], suggesting that other factors besides hyperglycemia can contribute to the alterations in platelet functionality. Already in 2001, Assert et al. [18] could show that short-term hyperglycemia without overt T2DM could lead to the activation of PKC in platelets, suggesting that glucose itself drives platelet activation. In 2003, Keating et al. [22] demonstrated that glucose in healthy subjects was able to induce platelet aggregation and to potentiate the effect of ADP, suggesting that not long term but rather acute alterations due to increased glucose concentrations can alter platelet functionality. In line with this, it has been recognized that platelets can also become activated in the postprandial phase in insulin resistant or diabetic patients but not healthy individuals, which is thought to be related to the extent of hyperemia [23, 24]. Interestingly though, a recent study in T1DM and T2DM individuals, which analyzed postprandial platelet activation, could demonstrate that this effect was only observed in T2DM subjects, suggesting that insulin is involved in the platelet activation events [25]. Another recent study, which compared platelet properties in T1DM vs T2DM patients [26], could show a decrease in platelet count and mass in T2DM vs T1DM patients. Taken together, there seems to be a correlation between increased plasma glucose and/or high spikes in plasma glucose, as observed in T1DM and T2DM, low insulin levels or decreased insulin sensitivity, and a higher propensity for platelets to become activated and blood clot formation, suggesting a link between these conditions.

## Insulin signaling in endothelial cells

Given that the key defining parameters of the MS are cardiovascular impairments in conjunction with insulin resistance, and since people suffering from the MS often have chronically elevated plasma insulin levels, it is worthwhile to examine the role of insulin in healthy endothelium. Besides the well-described role of insulin in glucose uptake via GLUT4 translocation in its target tissues muscle and adipose tissue, insulin also plays an important role in vascular function, in particular as a vasodilator [27]. The insulin receptor (IR) is expressed on endothelial cells, and endothelial-specific knockout of IR causes impairments in regulation of blood pressure and vascular tone, even though no major effects on glucose homeostasis were reported [28]. Insulin exerts its vasodilatory role primarily via modulation of nitric oxide (NO) production. NO, a free radical, which contributes to oxidative stress, is an important vasodilator released by endothelial cells that can diffuse through the plasma membrane and activates soluble guanyl cyclase leading to increased cGMP levels, which in turn promotes vascular smooth muscle relaxation [29]. Fitting with this vasodilatory role, NO also has a pronounced inhibitory effect on platelet activation [30]. Besides this, it is involved in immune cell function, where it is utilized as a toxic radical, and as a neurotransmitter, functions not covered here. NO is produced by the action of nitric oxide synthases (NOS), which as dimers catalyze the reaction of L-arginine with O_2_ to L-citrulline and NO, by accepting electrons from NADPH via the cofactor tetrahydrobiopterin (BH_4_) [31]. In an AKT-dependent manner, insulin causes the phosphorylation of endothelial nitric oxide synthase (eNOS) at S1177 (1178 in humans), leading to increased NO synthesis and release [32, 33]. This vasodilatory action of insulin seems to be functionally linked to insulin’s ability to reduce blood glucose, since several groups have shown that rate of muscle perfusion modulates glucose uptake and that insulin’s vasodilatory action efficacy correlates with its ability to elicit glucose clearance [34–36]. From a systemic standpoint, endothelial insulin action also plays an important role in delivering insulin to its target tissues. Originating from the pancreas, in order to reach its target cells, insulin must pass the endothelium. This trans-endothelial transport of insulin has been studied primarily in the context of muscle [37–39], and brain [40–42], and has been shown to be saturable, dependent on the insulin receptor, and to involve AKT signaling and insulin receptor endocytosis.

There is evidence that a variety of impaired endothelial functions precede the occurrence of pronounced systemic insulin resistance and overt T2DM. Among the reported decreased insulin signaling events is endothelial insulin transport, which has been shown to be decreased in the brains of dogs after 5 weeks of a high-fat diet [43]. In terms of muscle insulin signaling, there are mixed reports. While some groups describe reduced insulin transport through the endothelium contributing to decreased insulin action at the level of muscle cells [44], others report that trans-endothelial transport is not limiting in muscle insulin signaling [45]. Nonetheless, while impaired insulin transport to the muscle might play a role in insulin resistance, direct impairment at the level of the receptor in the muscle has been unequivocally established [46].

While long-term exposure to obesogenic stimuli and the low level of inflammation can cause insulin resistance in all insulin responsive tissues, the question whether endothelial insulin resistance might occur independently or indeed be a driver for systemic insulin resistance remains unanswered. One key paper could show that endothelial insulin resistance, as measured by insulin’s ability to stimulate eNOS phosphorylation, occurs weeks before detectable systemic and in particular muscle or adipose tissue insulin resistance [47]. If one considers endothelial insulin resistance as an early event in the development of the MS, it will remain to be resolved how ingestion of a western diet can impair endothelial insulin action.

## Post-prandial effects on NO-dependent vasodilation

Platelet reactivity and increased coagulation increase in concert with systemic insulin resistance, but are also observed acutely after ingestion of a high calorie meal in insulin resistant individuals. Similarly, impairments in vascular reactivity can be observed immediately after a meal. The most frequently used readout to assess postprandial vascular impairment is flow mediated vasodilation (FMD), commonly measured in the brachial artery. Changes in shear stress cause blood vessels to acutely release NO, which in turn causes a widening of the blood vessel. To determine FMD, a cuff is applied to the arm with increased pressure, which is released, and brachial artery diameter is continuously measured using ultrasound imaging. Alternatively, the direct effects of various vasoactive substances distinct from NO infused into the artery can be measured, but this technique is used less frequently than the noninvasive FMD [48, 49]. A large number of studies suggest that obese or diabetic individuals have stronger decreases in postprandial FMD than healthy individuals; however, it should be noted that overall postprandial effects on FMD are also observed in healthy people. It remains unclear which factors induce these postprandial effects and both glucose and lipids have been suggested as mediators, since circulating concentrations of both are elevated in the post-prandial state. Alternatively, hormones released in response to food intake could lead to altered FMD. Indeed, independent effects of FMD could be demonstrated for direct intravenous infusion of both glucose and lipids, suggesting that elevated blood levels of these nutrients can have direct effects on the endothelium [50, 51]. From a physiological perspective, it seems likely that lipids are the more important mediators of FMD, since various studies show that ingestion of high fat, as opposed to low-fat meals, causes impaired FMD, postprandially [52]. Interestingly, there seems to be a relation between the type of fat ingested and vascular reactivity, as long-chain, saturated fatty acids, which are thought to be the main mediators of inflammation, show more pronounced effects on vascular reactivity [53].

In agreement with the data on obesity and T2DM, there is also evidence that ingestion of high-fat meals has a cumulative effect on vascular function, with impairments becoming more pronounced and perhaps persisting over time [54, 55]. Conversely, exercise can acutely and persistently improve postprandial FMD, suggesting that the vasculature can adapt to metabolic stressors to modulate its responses acutely and in the long term [56].

## Vascular NO availability in obese and diabetic states

Besides the acute effects observed after ingestion of a HFD meal on FMD, which are mediated by eNOS activity and NO release, there is also a clear connection between obese and diabetic states, and reduced NO signaling [57]. Reduced NO bioavailability has been reported in both rodent models [47, 58, 59] and humans [60–62], but some groups also report increased NO production in obese humans [63, 64]. Various mechanisms are thought to contribute to this reduced NO availability, such as eNOS substrate availability, eNOS activity and levels, and NO stability, which is influenced by its high reactivity and dependent on the level of reactive oxygen species (ROS) [65]. In terms of substrate availability, it has been reported that plasma arginine levels are not acutely changed in early obesity in humans [66]. However, endothelial uptake of arginine can be impaired in conditions of inflammation, as typically observed in the vasculature of obese patients [67]. Moreover, there is a lot of evidence that the enzymatic activity of eNOS is changed in states of obesity or diabetes [57]. eNOS forms functional dimers in the presence of both ample L-arginine and BH_4_, a state called “coupled.” Various conditions such as lack of BH_4_ or high levels of arginase can cause eNOS to become uncoupled, a state in which it generates superoxide (O_2_^−^), a highly potent ROS, instead of NO. Interestingly, eNOS uncoupling is frequently observed in the endothelium of obese animals [68–70]. Another factor influencing eNOS activity is by competitive inhibition of alternative binding substrates, the most important of which is asymmetric dimethyl arginine (ADMA) [71]. Plasma levels of ADMA are known to be increased in obese or diabetic states and have been reported to correlate with impaired endothelial function [72–74]. In addition, eNOS-mediated NO production is dependent on the amount of eNOS present in the cell, and there have been reports of reduced eNOS levels in both muscle and adipose tissue of obese patients [75–77]. A further mechanism which could influence NO bioavailability, namely increased oxidative stress in the vascular endothelium, will lead to reduced NO levels through chemical reaction. This process is intricately connected to vascular disease in general, and impaired eNOS function, in particular. There are various sources of ROS in the vascular endothelium, besides the already mentioned action of uncoupled eNOS, which mostly contributes to ROS production in a state of chronically impaired endothelial function. These include the specialized ROS-producing NADPH oxidase (NOX) enzymes, enzymes of the mitochondrial respiratory chain, and xanthine oxidoreductase [65, 78, 79]. Endothelial ROS typically are produced as the superoxide anion O_2_^−^, which is converted to H_2_O_2_ by superoxide dismutase (SOD), which can be further degraded by catalases or peroxidases. Of these, superoxide is the most damaging and will rapidly react with NO to form peroxynitrite (ONOO^−^), a precursor of various reactive nitrogen species [80]. It should be noted that ROS also fulfill a relevant signaling function in healthy vasculature, and it is when their production becomes excessive in various disease states, that impairments and oxidative damage occurs [81, 82].

The fact that endothelial NO availability and vascular NO signaling are reduced in states of obesity and diabetes has prompted interest in attempting to correct the disease state by modulating NO availability or signaling. One popular mode of increasing NO availability is supplementation with L-arginine or L-citrulline, which have been consistently shown to have beneficial effects on overweight and insulin resistance. In rats, L-arginine supplementation decreased fat mass and insulin levels in both genetic and dietary obesity models [83, 84]. In mice, mild metabolic improvements due to L-arginine supplementation were reported in animals on a low-protein diet [85]. In humans, several studies attest to the efficacy of reducing abdominal fat and increasing insulin sensitivity following arginine supplementation, although effects are usually mild [86–90]. Modulating NO signaling downstream, which was achieved by long-term treatment with sildenafil, an inhibitor of the phosphodiesterase 5, which degrades cGMP and thereby turns off the vasodilatory signal of NO, attenuated body weight gain and improved plasma glucose levels and insulin signaling in mice on a HFD [91]. In a human trial, it was shown that 3 months of sildenafil treatment increased insulin sensitivity as assessed by hyperglycemic clamps in prediabetic subjects [92]. On the level of the enzyme eNOS, transgenic overexpression in mice was shown to increase metabolic health, and protect from HFD-induced obesity [93], and genetic variations of eNOS are associated with T2DM and aspects of the MS in humans. Overall, there is a clear correlation between reduced NO production and signaling, and obese and diabetic states, along with evidence that increasing these signals can have beneficial effects in both cases.

## Temporal dynamics and mediators of obesity-associated vascular impairments

While the associations of vascular impairments, obesity, and the MS are very clear, the causality, directionality of the associations, and temporal dynamics are far from understood. In the previous parts of this review, we have focused on platelets, clotting, and vascular NO-signaling as components of MS-associated impairments that show disturbances both acutely after a meal challenge as well as increasingly during the progression of the disease. There is a large variety of mediators and cytokines contributing to the progression of vascular disease in the context of obesity, but the two key plasma parameters that seem to be causal, both in the early postprandial phase, as well as in the long-term progression through persistent reappearance are FFAs and glucose. In prediabetes, fasting glucose levels as well as postprandial spikes are higher than in healthy subjects [94], whereas FFA levels are elevated strongly during fasting and drop less after a meal compared to healthy individuals [95]. As has been discussed previously, high peaks of plasma glucose have been shown to be able to activate platelets, and chronically elevated plasma glucose can lead to the formation of AGEs, which have been functionally linked to endothelial dysfunction. One should consider, however, that chronically elevated plasma glucose levels typically only occur very late in the context of the MS, which makes the frequency and magnitude of glucose peaks a more likely immediate cause for the described hemostatic and vascular effects than AGEs. While acute high-glucose loads have an effect on both platelet activation and endothelial function, it is typically FFAs which are the predictors of platelet activation. There is a large consensus that meals high in lipids, causing elevation of plasma FFA, are causal in the generation of both vascular disease and T2DM. Along this line, FFA alone can cause decreased NO production in endothelial cells [96], and HFD feeding causes insulin resistance in the endothelium of the aorta (an ostensibly other endothelia) several weeks before the appearance of insulin resistance in adipose tissue and muscle [47]. Furthermore, several clinical trials assessing the effects of good glucose control in diabetics on CVD demonstrate that benefits are modest, reporting decreases in microvascular risk and small decreases of all-cause mortality over 10 years, suggesting that glucose alone is not sufficient to explain these effects [97, 98]. One possible link for both glucose and FFA in relation to T2DM development and CVD is reactive oxygen stress. Both glucose and FFA can elicit an increase in ROS, in vitro and in vivo, acutely and chronically [99–102]. Similarly, oxidative stress has also been linked to the complications occurring in T1DM [103, 104]. Importantly, there is a very clear causal relation between ROS formation and the activation of various inflammatory pathways, in particular of the innate immune system [105]. Besides ROS, it also has been demonstrated that FFA can directly activate the inflammatory NFkB-pathway through TLR4, and both of these pathways have been frequently described to be involved in the development of the MS [106, 107]. This allows the establishment of a putative causal chain from endothelial disruptions and hemostatic events caused by increased ROS due to excess FFA and glucose, leading to progressive inflammation, which eventually causes insulin resistance of the main insulin responsive organs, as well as the vascular damage that predisposes to cardiovascular complications.

## Inflammation as the common denominator for cardiovascular disease and the metabolic syndrome

In discussing the connections between the MS and T2DM, it is important to remember that both of these diseases have a relevant and causal involvement of inflammation and could therefore be said to be inflammatory diseases. In the case of CVD, the key risk factor observed in patients, beside elevation of blood pressure and elevated plasma lipids, is atherosclerosis, a thickening of the blood vessel wall due to invasion of leukocytes containing lipids [108]. While atherosclerosis was originally considered a disease caused by impaired lipid storage, it has since been convincingly demonstrated that it is actually a result of misguided inflammatory processes [109]. At the same time, insulin resistance in the context of T2DM was originally thought to be due mainly because of metabolic alterations. Because of the seminal work of Hotamisligil et al. [110], demonstrating the release of the pro-inflammatory cytokine TNFα from obese adipose tissue, it became clear that inflammatory cytokines from adipose tissue due to a chronic low-level inflammation of the tissue are fundamental contributors to the MS and T2DM. A lot of work exists demonstrating that preventing or ameliorating inflammation in adipose tissue prevents insulin resistance and/or weight gain in the context of diet-induced obesity [111]. However, the root cause of adipose tissue inflammation is still incompletely understood. One of the original ideas, apoptosis of macrophages in adipose tissue due to metabolic stress, did not withhold scrutiny and current front-runners are endoplasmatic reticulum- stress or hypoxia [112].

Considering the evidence described in this review, we propose that a cause of the inflammation of adipose tissue, and probably also the low-level inflammation in observed in other tissues in the MS, is the damaged and modified endothelium in concert with the fact that platelets become hyperactive. Several papers, besides the already mentioned work demonstrating that endothelial insulin resistance precedes systemic insulin resistance [47], illustrate that endothelial activation precedes inflammation and immune cell invasion of adipose tissue. For example, Nishimura et al. showed that in obese and HFD-fed mice, there is increased platelet-leukocyte interaction and tethering, and blocking the endothelial inflammatory surface marker ICAM1 can normalize these effects [113]. Cleuren et al. showed that HFD feeding leads to a rapid increase of circulating blood clotting factors, and the source of these is the activated endothelium rather than the liver, which is the main source of circulating clotting factors [114]. In addition, studies interfering with basic activators of blood clotting or platelet ligands have been shown to ameliorate HFD-induced inflammation and insulin resistance, such as mice lacking PAR2, a key target of tissue factor/factor VII mediated coagulation, or mice lacking the PSGL1, a main ligand for P-selectin, a surface protein exposed on activated platelets [115, 116]. Moreover, there is strong evidence that treatment with n-3 fatty acids or the prostaglandin synthase 2 inhibitors aspirin or salsalate, which have been shown to have both anti-inflammatory, as well as anti-coagulatory effects, is beneficial in settings of obesity and the metabolic syndrome [23, 24, 117, 118].

In conclusion, in this review we provide a broad overview of changes in hemostasis and endothelial function occurring in obesity, the MS, as well as the acute postprandial phase. These impairments converge on oxidative stress and pro-inflammatory signals in the endothelium, which have a causal, and bidirectional link to insulin resistance, suggesting that endothelial activation and insulin resistance is one driver underlying both cardiovascular as well as metabolic impairments, occurring as a result of chronic ingestion of a western-type diet.
